# State of Health Evaluation of Lithium-Ion Batteries Using the Statistical Properties of the Voltage

**DOI:** 10.3390/e28020221

**Published:** 2026-02-14

**Authors:** Abdelilah Hammou, Raffaele Petrone, Demba Diallo, Claude Delpha, Hamid Gualous

**Affiliations:** 1 LUSAC Laboratory, University of Caen Normandy, 50130 Cherbourg-en-Cotentin, France; abdelilah.hammou@unicaen.fr (A.H.); raffaele.petrone@unicaen.fr (R.P.); hamid.gualous@unicaen.fr (H.G.); 2 GeePs Laboratory, CentraleSupelec, CNRS, University of Paris-Saclay, 91192 Gif-sur-Yvette, France; 3 L2S Laboratory, CentraleSupelec, CNRS, University of Paris-Saclay, 91192 Gif-sur-Yvette, France; claude.delpha@lcentralesupelec.fr

**Keywords:** lithium-ion batteries, state of health, statistical properties, Kullback–Leibler divergence, dynamic profile

## Abstract

Conventional indicators of battery health, such as capacity and energy, are difficult to measure directly and are therefore often estimated. This article proposes assessing lithium-ion battery health using the statistical properties of the voltage across the battery terminals, a measurement already available in battery management systems. The evolution of the voltage probability density function during the cycle is assessed using Kullback–Leibler divergence (KLD) as a health indicator. It is studied for two battery chemistries (Lithium iron Phosphate (LFP) and Nickel Manganese Cobalt (NMC)). The batteries are subjected to cycles with a dynamic current profile derived from globally harmonised test cycles for light vehicles (WLTC). Spearman’s correlation coefficients, above 86% for NMC cells and 74% for LFP cells, also indicate that this new health indicator is strongly correlated with conventional measurements of battery health (capacity or energy). The analysis also shows that the divergence not only closely follows the degradation trend even at high noise levels (SNR = 10 dB) but is also insensitive to noise levels higher than 30 dB.

## 1. Introduction

Lithium-ion batteries are among the most promising solutions for energy storage in electric vehicles due to their higher energy and power density [1,2]. However, the efficiency and safety of these energy storage systems depend on their state of health (SoH) [3,4]. Thus, online monitoring of battery cell health is essential and should be integrated into the battery management system. In the literature, several studies have been conducted on the estimation of lithium-ion battery state of health. These works focus on estimating the physical parameters of the battery correlated with battery ageing, such as available capacity, energy, and internal resistance [5,6,7]. The state of health estimation methods can be divided into two categories: physics-based methods and data-driven methods [5,8].

Physics-based approaches (also called model-based) use mathematical/physical models that mimic lithium-ion battery ageing. The objective of these methods is to estimate the model parameters based on battery measurements. Therefore, the evolution of the model parameters with ageing allows battery health supervision. In [9], the authors used a multithreaded dynamic optimisation method for state-of-health and state-of-charge estimation based on a model that combines fractional-order and Gaussian linear models. The method’s effectiveness is verified experimentally using the data from four battery cells. The approach presented in [10] used an enhanced electrochemical model to co-estimate the battery capacity and state of charge. The efficacy of these methods is strongly related to the accuracy of the battery’s model and the performance of the estimation algorithms.

Data-driven approaches use experimental ageing data to extract health indicators from available measurements for the SoH monitoring [11,12,13,14,15]. Most methods estimate battery capacity or resistance [11]. These methods require available battery measurements (voltage, current, temperature) along with corresponding health indicators to develop and fit the diagnostic model [12,13]. Recently, several data-driven algorithms have been proposed for SoH estimation, such as recurrent neural networks (RNNs), autoencoders (AEs), transformers [14], and extreme learning machine (ELM) [15]. In [15], the battery capacity is estimated using an ELM-based capacity model which parameters are optimised with the particle-swarm algorithm. The validation is conducted with experimental degradation data under several conditions. The authors in [16] propose a bidirectional Long Short-Term Memory (LSTM) network with an attention mechanism for SoH estimation. The features are extracted under constant battery current discharge. The method was validated under two different current profiles. In [17], the authors proposed a temporal conventional network with transfer learning to estimate capacity using the ampere-hour method and Monte Carlo simulations. The method was validated using the NASA dataset and a real-world vehicle dataset. The results show the effectiveness of the transfer learning in improving estimation accuracy. In [18], the study proposed a transformer network for capacity estimation. The accuracy and robustness of the method are enhanced through data augmentation with a time-series generative adversarial network (GAN). The method’s accuracy is validated on the Oxford dataset.

The discussed approaches presented accurate and robust results. However, these proposals require measuring or estimating the battery’s capacity or energy to assess SoH, which limits their applicability under varying operating conditions and with limited computational resources [19]. The proposal developed in this work addresses the challenge of estimating the battery’s state of health solely from the voltage measured at its terminals. The new health indicator exploits the evolution of the statistical properties of the battery’s voltage over its life cycle. More specifically, the health indicator measures the distortion of the voltage probability density functions during dynamic cycling. Through experimental tests, two lithium battery chemistries (Lithium iron Phosphate (LFP) and Nickel Manganese Cobalt (NMC)) are cycled using WLTC current profile. The measured data are used to evaluate the efficiency of the proposed health indicator based on the Kullback–Leibler divergence (KLD). The paper also analyses the relationship between this new health indicator and the usual measures of the state of health (capacity and energy).

The rest of the paper is organised as follows: Section 2 details the experimental ageing tests and introduces the Kullback–Leibler divergence. Section 3 presents the degradation results, the evolution of voltage measurements and KLD calculations with ageing, correlation analysis, and the robustness of KLD to measurement noise. A conclusion closes the paper.

## 2. Materials and Methods

### 2.1. Experimental Ageing Tests

**Figure 1:**
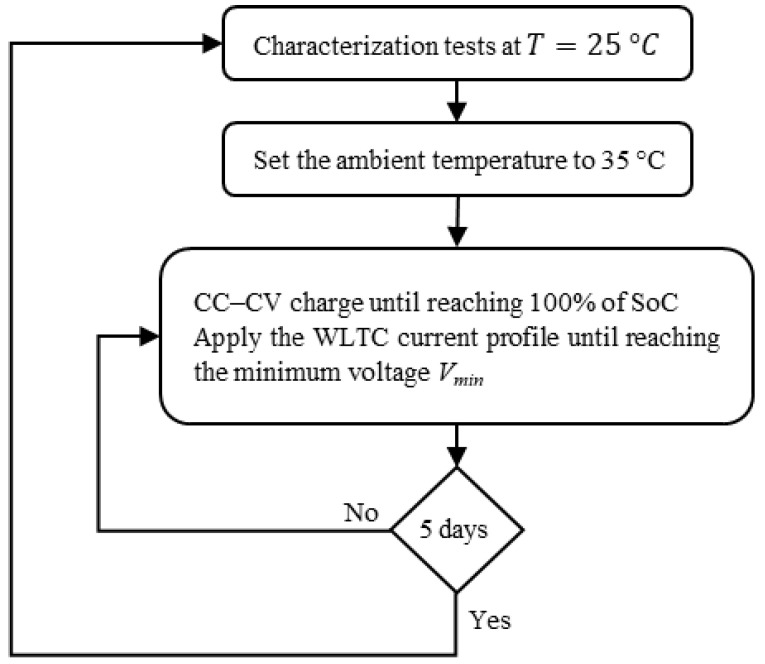
Experimental procedure for battery cycling tests.

**Figure 2:**
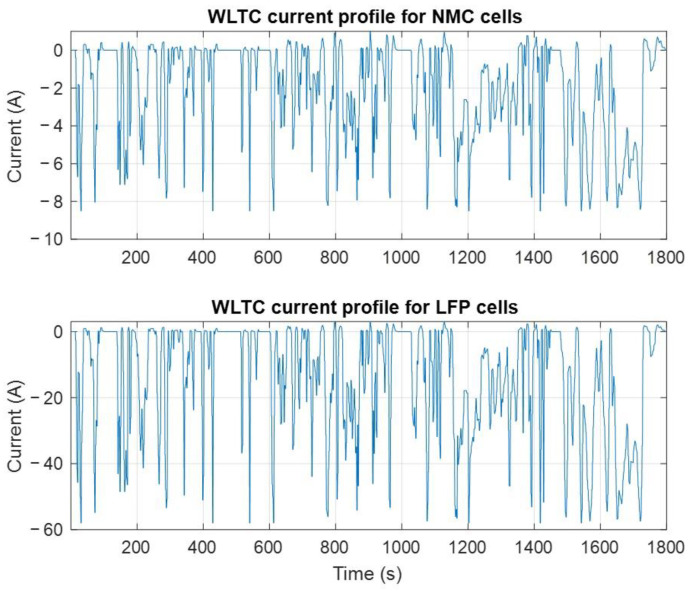
The used WLTC current profiles.

The battery test is carried out at a constant, moderate temperature of T = 35 °C to gradually accelerate ageing without causing irreversible damage [20]. The flowchart in Figure 1 outlines the electrical cycling of lithium-ion cells. The cells are first charged up to 100% state of charge (SoC) using CC-CV charge. Then, the cells are discharged by repeatedly applying the WLTC [19] current profile (Figure 2) until the cell’s voltage reaches its minimal value (cut-off voltage). The charge and discharge are replicated for five days. Then, a characterisation test is performed at the room temperature of 25 °C to evaluate each cell’s performance.

#### 2.1.1. Characterisation Tests

The characterisation test aims to measure the battery’s capacity, energy, and direct current internal resistance (DCIR). The capacity and energy of the cells are obtained from current and voltage measurements during the discharge of the cells with a constant current of C/5, as indicated by the following equations:

(1) $$C\left(Ah\right)=\int_{0}^{t_{end}}i\left(t\right).dt$$

(2) $$E\left(Wh\right)=\int_{0}^{t_{end}}i\left(t\right)U_{batt}\left(t\right).dt$$

where $t_{end}$ is the discharge time, and *U_batt_* the cell’s terminal voltage.

The DCIR of the cell is calculated from the voltage response to a current pulse of 1C during 30 s at 25 °C with an SoC of 100% [21].

(3) $$DCIR\left(\Omega \right)=\frac{\left(U_{ocv}-U_{1\mathrm{batt}}\right)}{I_{pulse}}$$

Where $I_{pulse}$ is the current pulse, $U_{ocv}$ is the measured Open Circuit Voltage (OCV), and $U_{1batt}$ is the cell voltage measured at the end of the pulse.

#### 2.1.2. Description of the Testbench

**Figure 3:**
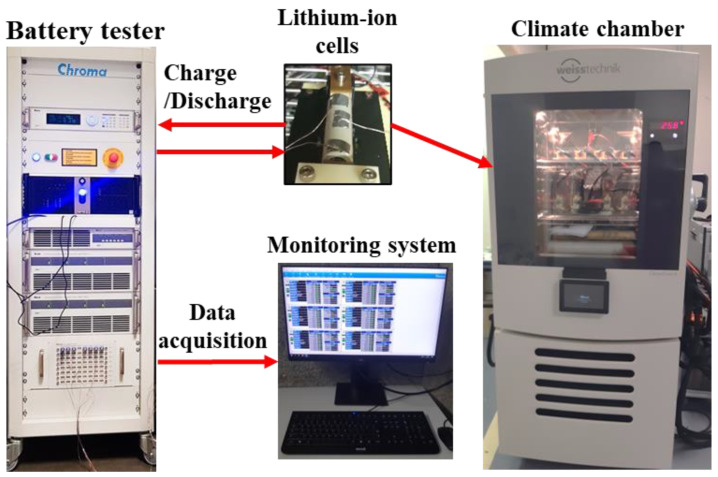
Experimental setup for battery ageing tests.

The tests are realised using the experimental testbench presented in Figure 3. It is composed of the following:

- Battery cycler: This device allows battery cycling using several modes: constant current, constant voltage, constant power, and dynamic current profile. It allows for cell supervision and the acquisition of battery cell terminal voltage, applied current, and temperature at various points on the cell surface.
- Climate chamber: This climatic chamber sets the thermal condition of the batteries: electrical cycling at 35 °C and check-up at 25 °C.
- Lithium-ion cells: The setup contains three cylindrical cells of nickel manganese cobalt chemistry/graphite (NMC) and four cells of lithium iron phosphate/graphite (LFP) battery are cycled [22]. Table 1 summarises the cells’ characteristics:

### 2.2. Introduction of the Kullback–Leibler Divergence (KLD)

**Figure 4:**
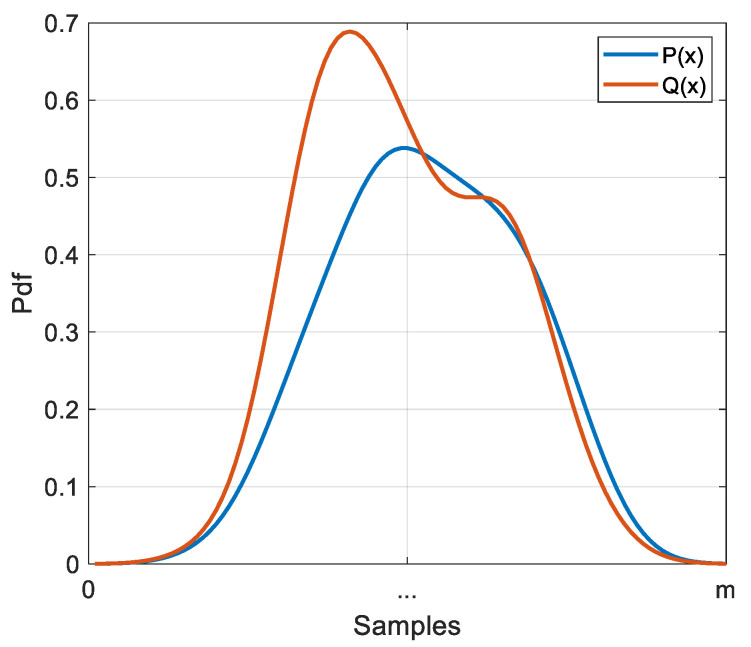
A representation of two probability density functions P and Q.

The health status of a battery cannot be measured directly. It is therefore estimated based on measurements available during its operation. This study focuses on the evolution of the global statistical properties of voltage under a dynamic current profile (WLTC). The proposed health indicator is based on the measurement of the distance between the probability distributions of voltage measurements at the battery terminals during ageing as displayed in Figure 4. There are several tools to measure the distance (or dissimilarity) between information measures [23]. It has been demonstrated that Kullback–Leibler divergence (KLD) is a measure suited to practical applications [24,25] and that it also constitutes an upper bound for several distance measures [26], making it more sensitive to slight variations.

Considering two continuous probability density functions *f* and *g* of a random variable *x*, the KLD (symmetrical version of the Kullback–Leibler Information) [27] is expressed in (4):

(4) $$KLD\left(f,g\right)=\int f\left(x\right)log\frac{f\left(x\right)}{g\left(x\right)}+\int g\left(x\right)log\frac{g\left(x\right)}{f\left(x\right)}$$

For its application to the battery case, the discretized version of the KLD is as follows in (5):

(5) $$KLD\left(P_{i},\;P_{BoL}\right)=\sum_{i=1}^{m}(P_{i}-P_{BoL})log(\frac{P_{i}}{P_{BoL}})$$

Where m is the number of samples, and $P_{BoL}$ and $P_{i}$ are the estimated probability distributions of the voltage measurements at the first discharge cycle (BoL) and at the *i*th cycle, respectively.

## 3. Results

### 3.1. Ageing Experiments Results

The capacity and energy fade, as well as the growth of the resistance, are calculated relative to their initial values at the beginning of life (BoL) using Equations (6), (7), and (8), respectively.

(6) $$SoH=Capacity\_f\left(\%\right)=100\frac{C_{i}}{C_{BoL}}$$

(7) $$Energy\_f\left(\%\right)=100\frac{E_{i}}{E_{BoL}}$$

(8) $$DCIR\_g\left(\%\right)=100\left(\frac{DCIR_{i}}{DCIR_{BoL}}-1\right)$$

Where $C_{BoL}$, $E_{BoL}$, $DCIR_{BoL}$, and $C_{i}$, $E_{i}$, $DCIR_{i}$ are measured at the beginning of life and at the cycle i, respectively.

**Figure 5:**
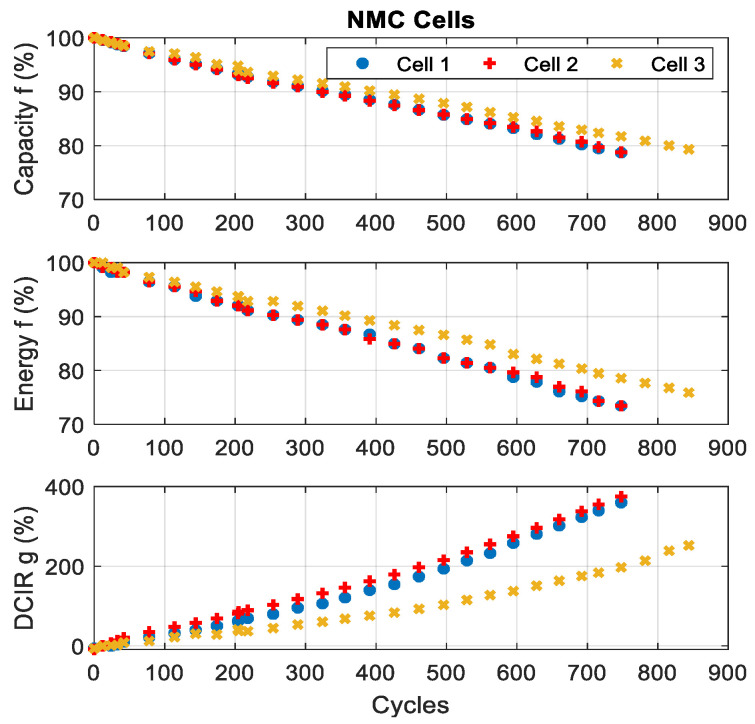
Results of characterisation tests for NMC cells.

**Figure 6:**
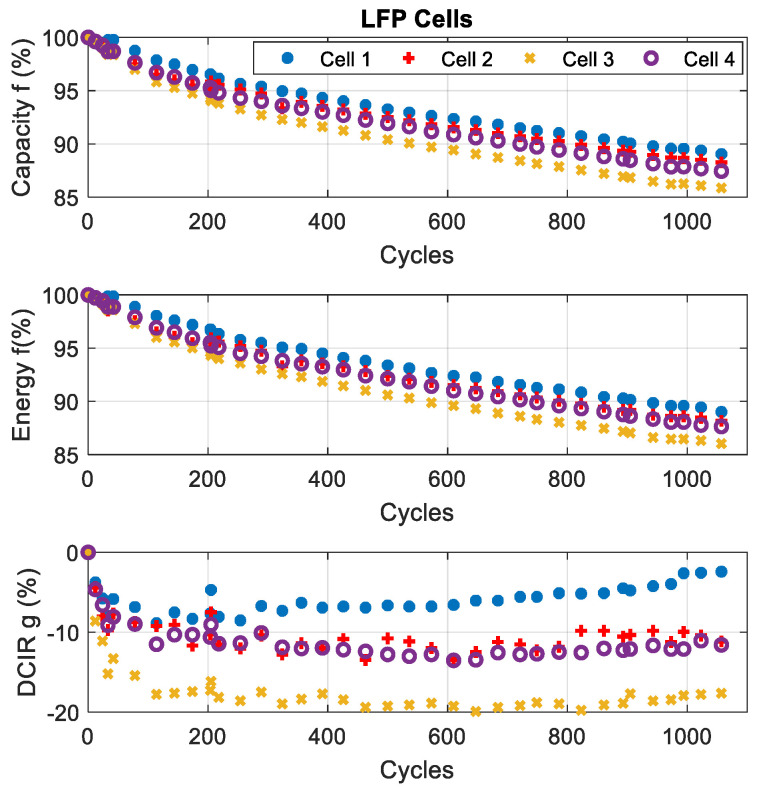
Results of characterisation tests for LFP cells.

The evolution of these battery parameters during cycling are displayed in Figure 5 and Figure 6. For both chemistries, the capacity and energy decrease with the increasing number of cycles:

- For the NMC cells, the capacity and energy fade by 21% and 27%, respectively (the worst reduction).
- For the LFP cells, the reduction in capacity and energy reaches 14%.

The evolution of the DCIR shows a significant increase in NMC cells (+350%), while in LFP cells, it decreases during the first 400 cycles before increasing.

The comparison shows that LFP cells have a longer lifespan than NMC cells. Indeed, NMC cells lose 20% of their capacity in less than 850 cycles, while LFP cells reach 1057 cycles with a capacity loss lower than 15% [28].

### 3.2. The Evolution of the Battery’s Voltage with Ageing

**Figure 7:**
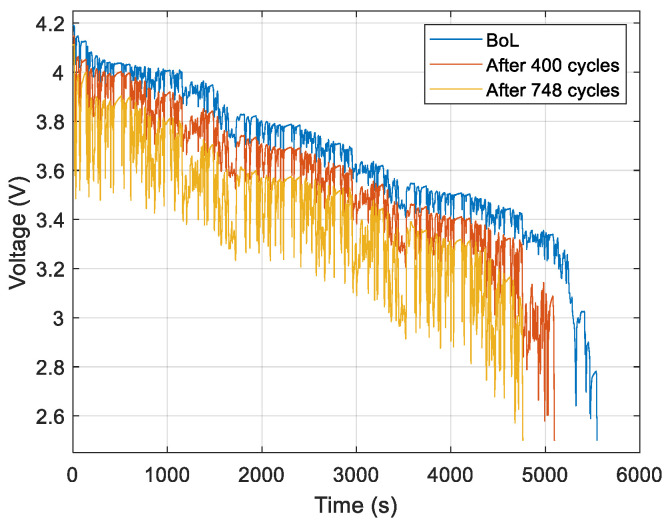
Voltage measurement for the NMC cell 1 under discharge conditions.

**Figure 8:**
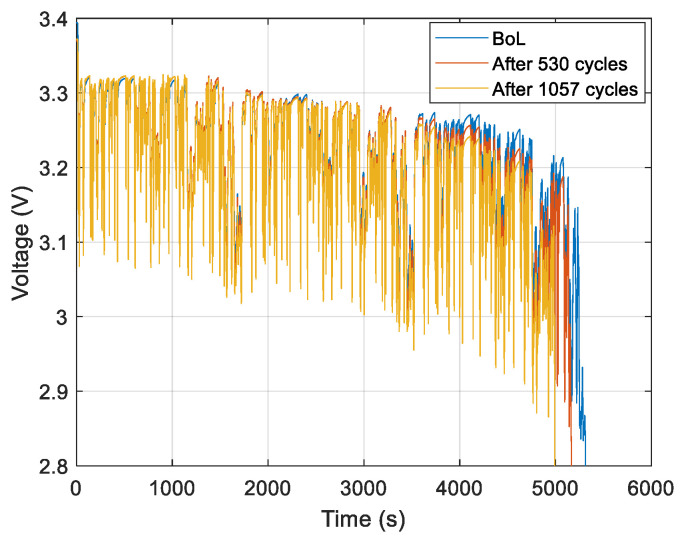
Voltage measurement for the LFP cell 1 under discharge conditions.

Figure 7 and Figure 8 show the measured voltages during battery discharge under the WLTC current profile for NMC cell 1 and LFP cell 1, respectively. The voltage waveforms are displayed for three cases: at the beginning of life (BoL), after 400 cycles, after 748 cycles for NMC cells, and at the BoL, after 530 cycles, and after 1057 cycles for LFP cells.

We can conclude from Figure 7 and Figure 8 that the voltage is affected by the ageing.

The discharge duration decreases with ageing due to capacity fade in both chemistries.

- For the NMC cells (Figure 7), the voltage decreases with ageing.
- For the LFP cells (Figure 8), the ageing effects are more significant at low SoC, as observed in the time interval between 3500 and 5300 s.

### 3.3. Application of Kullback–Leibler Divergence (KLD) to SOH Estimation

**Figure 9:**
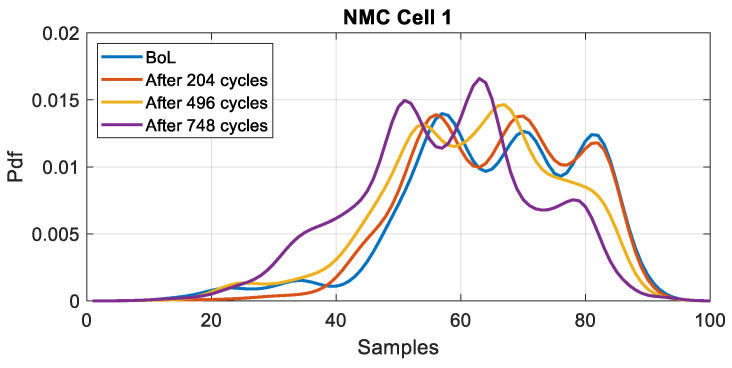
PDF of the voltage measurement at different ageing stages for NMC (cell 1).

**Figure 10:**
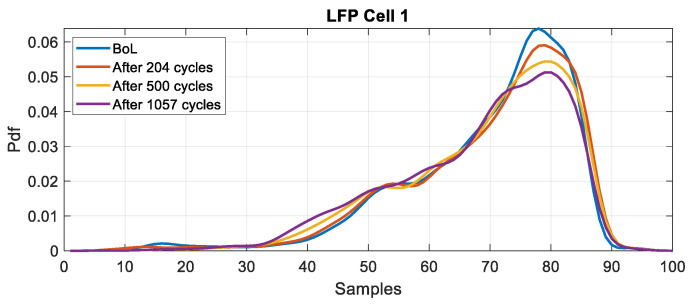
PDF of the voltage measurement at different ageing stages for LFP (cell 1).

The probability density function (pdf) of the battery voltage under discharge conditions, using the WLTC current profile, is calculated via Monte Carlo simulations [29]. The simulations are performed 50 times with Additive White Gaussian Noise (AWGN) at a Signal-to-Noise Ratio (SNR) of 50 dB. The PDF is estimated using the Gaussian Kernel (mean value = 0 and variance = 1). The sampling frequency for voltage measurement is set at $f_{s}=1\;\mathrm{Hz}$. The evolution of the PDF is plotted for different states of health in Figure 9 and Figure 10 for NMC and LFP cells, respectively. All probability density functions are estimated under the same load profile (WLTC) and over the same SoC range (100% to 0%).

**Figure 11:**
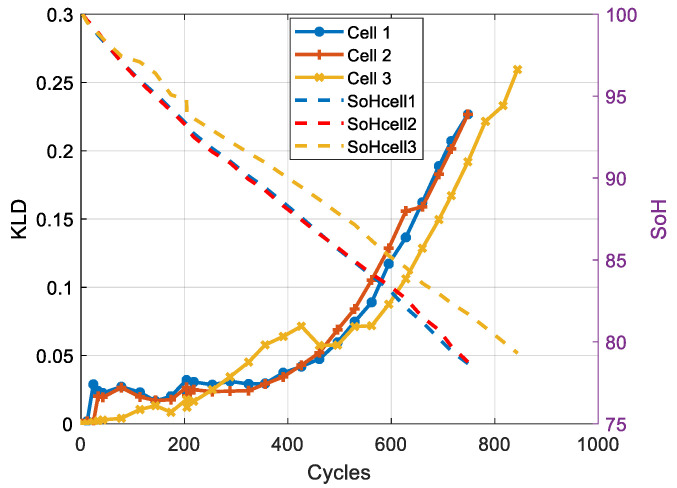
Evolution of the KLD and SoH for the three NMC cells.

**Figure 12:**
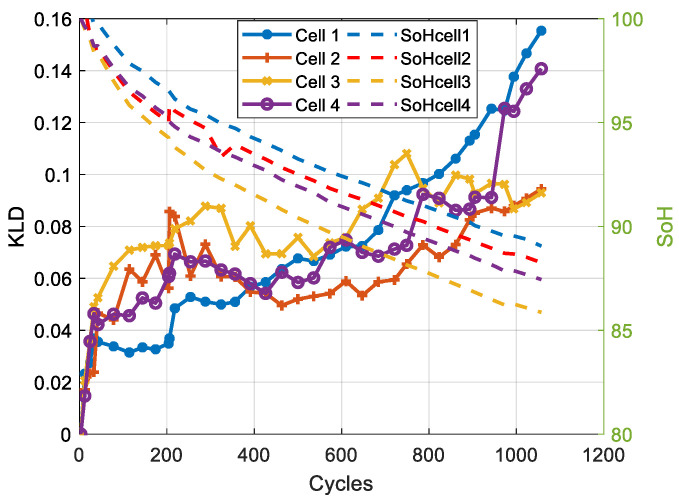
Evolution of the KLD and SoH for the four LFP cells.

The results show that for both chemistries, the PDF is affected by the ageing. For the NMC cell, we note a shift and a deformation of the peak. For the LFP cell, we observe a decrease in the peak value. Figure 11 and Figure 12 present the evolution of the KLD and the SoH as a function of the number of cycles for NMC and LFP cells, respectively.

The results presented in Figure 11 and Figure 12 show that, for both chemistries, KLD increases with the number of cycles. We also note that the evolution of KLD is non-linear compared to that of SoH, making this health indicator more representative of battery ageing, which is a non-linear phenomenon [30].

For the NMC cells, except at the beginning of life, two trends with distinct slopes can be distinguished over the life cycle: one for the first 500 cycles and one for the remainder of life (EoL).

### 3.4. KLD Performance Evaluation

#### 3.4.1. Correlation Coefficients

This section evaluates the correlation between the energy and the capacity of the battery cells with the KLD. The correlation is evaluated using Pearson’s and Spearman’s coefficients. The Pearson correlation [31,32] represents the linear dependency between two variables X and Y as described in (9):

(9) $$R_{Pearson}=\frac{1}{n}\sum_{i=1}^{n}\left(\left(\frac{X_{i}-\bar{X}}{\sigma_{X}}\right)\left(\frac{Y_{i}-\bar{Y}}{\sigma_{Y}}\right)\right)$$

The Spearman correlation [33,34] quantifies the existence of a monotonic linear or non-linear relationship between two variables. The Spearman correlation uses the ranks of the observations ($rank\left(X\right)$ and $rank\left(Y\right))$ to calculate the correlation score.

(10) $$R_{Spearman}=1-\frac{6\sum_{i=1}^{n}d_{i}^{2}}{n^{3}-n}$$

Where *n* is the number of observations, and $d_{i}$ the difference between $rank\left(X_{i}\right)$ and $rank\left(Y_{i}\right)$. Given the negative correlation between the evolution of battery capacity and energy and the evolution of the KLD (Figure 11 and Figure 12), the correlation coefficients are presented with their absolute values.

#### 3.4.2. Correlation Analysis for NMC Cells

**Figure 13:**
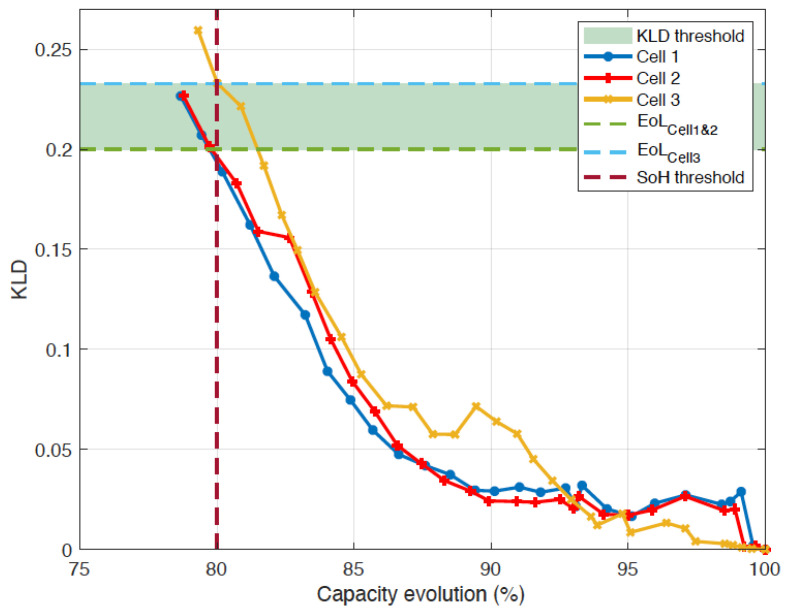
Evolution of the KLD versus capacity for NMC cells.

**Figure 14:**
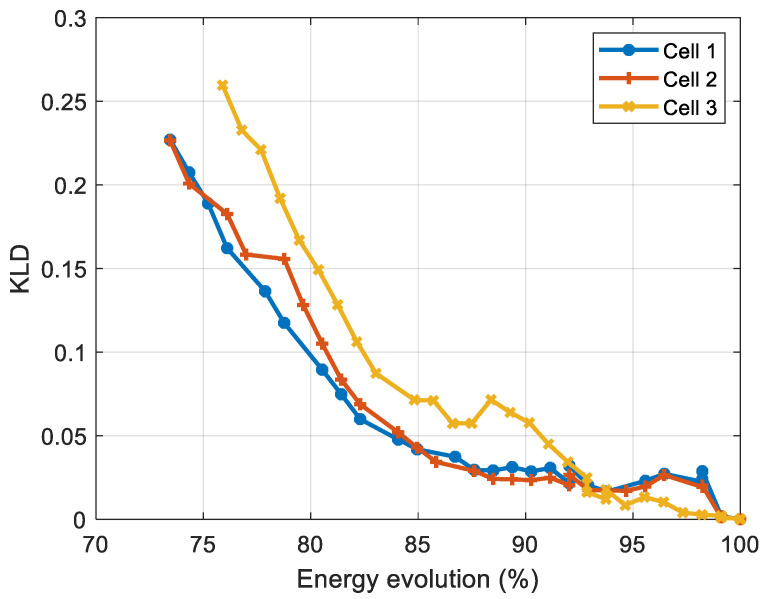
Evolution of the KLD versus energy for NMC cells.

Figure 13 and Figure 14 display, for NMC cells, the evolution of KLD as a function of capacity fade, and energy fade, respectively, while Table 2 and Table 3 present the correlation coefficients (absolute values) between KLD and capacity, and between KLD and energy, respectively.

The analysis of the correlation coefficients shows that the KLD obtained from the voltage measurements is strongly correlated with the cells’ capacity and energy. We also note that the three cells meet the end-of-life criteria based on capacity fade (a 20% loss of capacity) when the KLD lies between 0.20 and 0.23. The comparison of the two correlation coefficients shows that the Spearman coefficients are higher, indicating a non-linear relationship between the battery’s capacity and the KLD.

#### 3.4.3. Correlation Analysis for LFP Cells

**Figure 15:**
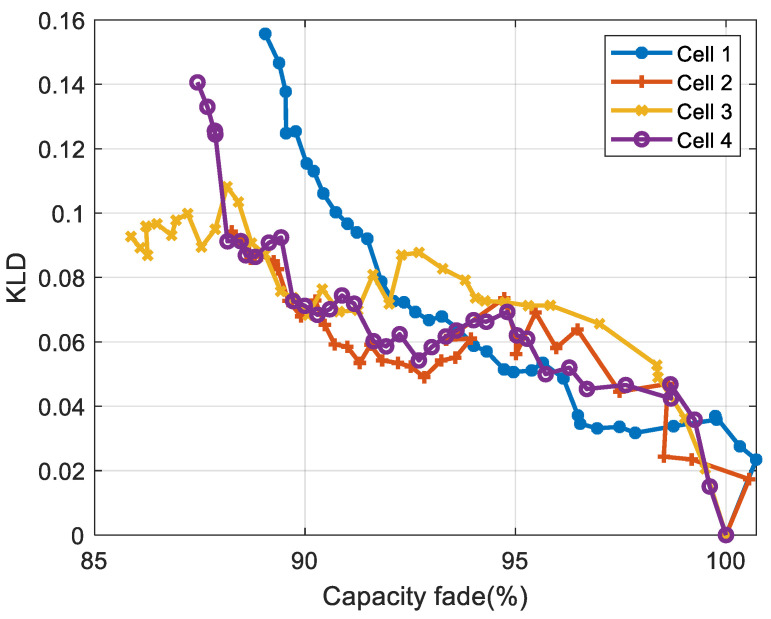
Evolution of the KLD versus capacity for LFP cells.

**Figure 16:**
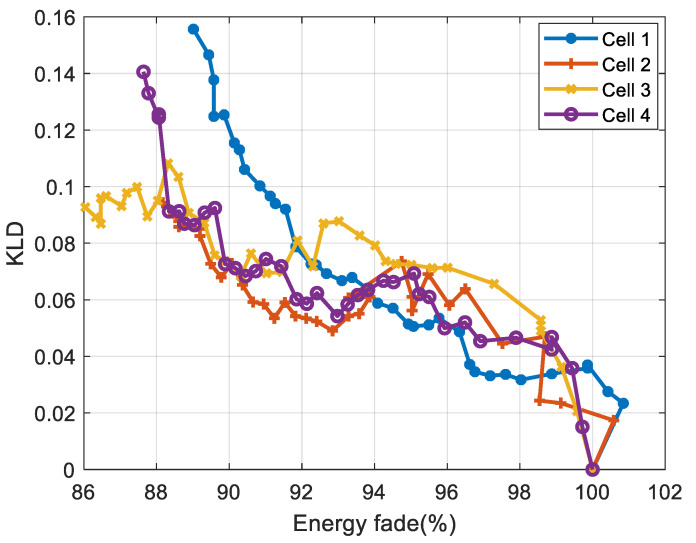
Evolution of the KLD versus energy for LFP cells.

Figure 15 and Figure 16 display, for NMC cells, the evolution of KLD as a function of capacity fade and energy fade, respectively, while Table 4 and Table 5 present the correlation coefficients (absolute values) between KLD and capacity, and between KLD and energy, respectively.

The results show that the KLD is strongly correlated with the cells’ capacity and energy for NMC cells. The comparison shows that Spearman correlation coefficients are higher than Pearson’s correlation coefficients, indicating a non-linear relationship between the KLD and the battery’s capacity and energy.

For LFP cells 1, 3, and 4, the correlation coefficients are higher than 83%. While for LFP cell 2, the correlation coefficient is lower: the Pearson correlation is around 74%, whereas the Spearman correlation is around 66%. This difference is due to the ageing inconsistency between cells, as reported in several studies [35]. LFP cell 2 exhibits aberrant ageing behaviour compared to the other cells, as shown in Figure 12. The lower Spearman coefficient reflects the irregular ageing behaviour of LFP cell 2, which introduces rank inversions and deviations from strict monotonicity. Since the Spearman coefficient is based on rank ordering (Equation 10), it is more affected by these disruptions. The Pearson coefficient captures the overall linear trend and therefore remains relatively high.

### 3.5. Robustness Analysis

**Figure 17:**
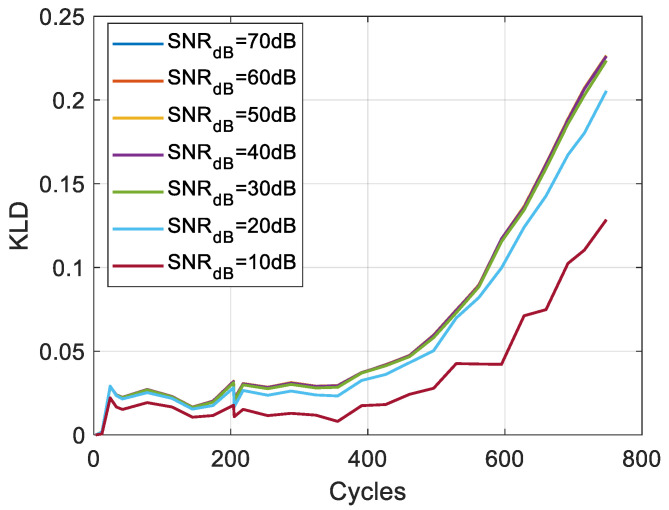
Evolution of the KLD for NMC cell 1.

**Figure 18:**
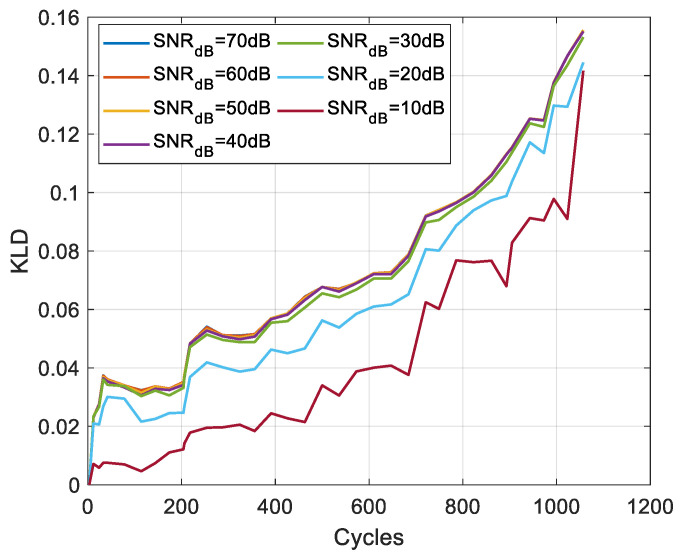
Evolution of the KLD for LFP cell 1.

To evaluate the robustness of the KLD to measurement noise, white Gaussian noise with a Signal-to-Noise Ratio (SNR) ranging from 70 to 10 dB is added to the voltage measurements. Monte Carlo simulations are realised 500 times to generate the PDFs and calculate the KLD. Figure 17 and Figure 18 present the results for one cell of each chemistry, respectively. The results show that the KLD is insensitive to the noise level for SNR above 30 dB. However, at higher noise levels, even with lower values, the KLD pattern remains consistent with the degradation trend.

From the experimental results and analysis, we can conclude that the KLD shows a high correlation with the usual health indicators, namely capacity and energy [11,12,13,14,15,16,17]. The proposed KLD used as a health indicator exhibits high robustness to noise in measurements, making it suitable for real applications where measurements are always affected by noise. Regarding the data requirements for method implementation, KLD extraction requires the calculation of the probability density function at the beginning of life (BoL), which is feasible since the battery management system provides the voltage measurements. Unlike machine learning and deep learning methods that require large datasets and long training and validation times, the proposed method does not require a large amount of ageing data to be designed and tuned.

## 4. Conclusions

This study introduces the Kullback–Leibler Divergence as a new health indicator for lithium-ion batteries. It is calculated as the distance between the probability density functions of the battery’s terminal voltage at the beginning of its life and the current cycle. The indicator’s performance is evaluated using experimental data from dynamic cycling of lithium-ion cells (LFP and NMC chemistries). The results show that the proposed health indicator monotonically increases with battery ageing. The results also showed a strong correlation between the KLD and the battery’s usual ageing health indicators (capacity and energy): the Pearson correlation coefficient is greater than 86% for NMC cells and 74% for LFP cells. The proposed health indicator only requires voltage measurements (at the beginning of life and at the current cycle). It also does not require large datasets. Furthermore, this health indicator shows high robustness to voltage measurement noise, making it a suitable technique for battery monitoring in industrial applications. Future work will focus on evaluating the KLD under different working conditions and scenarios, and on comparing it with other health indicators, such as internal resistance, incremental capacity peaks, and relaxation voltage characteristics.

## Figures and Tables

**Table 1:** Characteristics of the NMC and LFP cells.

Characteristics	NMC Cells	LFP Cells
Model Name	INR21700-30T	ZG-LFP020AH
Dimension	Diameter: 21 mm, Height: 70.1 mm	Width: 71 mm, Length: 178 mm, Height: 28 mm
Nominal capacity	3 Ah	20 Ah
Operating voltage	2.5 V to 4.2 V	2.8 V to 3.8 V
Max charge current	4 A	20 A

**Table 2:** The absolute values of capacity correlation coefficients for NMC cells.

Correlation Coefficient	Cell 1	Cell 2	Cell 3
Spearman	0.933	0.953	0.989
Pearson	0.866	0.883	0.927

**Table 3:** The absolute values of energy correlation coefficients for NMC cells.

Correlation Coefficient	Cell 1	Cell 2	Cell 3
Spearman	0.939	0.955	0.988
Pearson	0.882	0.898	0.937

**Table 4:** The absolute values of capacity correlation coefficients for LFP cells.

Correlation Coefficient	Cell 1	Cell 2	Cell 3	Cell 3
Spearman	0.987	0.668	0.832	0.934
Pearson	0.928	0.752	0.843	0.872

**Table 5:** The absolute values of energy correlation coefficients for LFP cells.

Correlation Coefficient	Cell 1	Cell 2	Cell 3	Cell 3
Spearman	0.987	0.668	0.831	0.933
Pearson	0.931	0.749	0.838	0.870

## Data Availability

The data will be available on request.
